# The membrane receptors that appeared before their ligand: The different proposed scenarios

**DOI:** 10.1371/journal.pone.0231813

**Published:** 2020-05-22

**Authors:** Anna Grandchamp, Philippe Monget

**Affiliations:** PRC, UMR85, INRA, CNRS, IFCE, Université de Tours, Nouzilly, France; Cleveland Clinic Lerner Research Institute, UNITED STATES

## Abstract

The interactions between membrane receptors and their endogenous ligands are key interactions in organisms. Recently, we have shown that a high number of genes encoding human receptors appeared at the same moment as their ligand in the animal tree of life. However, a set of receptors appeared before their present ligand. Different scenarios have been proposed to explain how a receptor can be conserved if its ligand is not yet appeared. However, these scenarios have been proposed individually and have never been studied in a global way. In this study, we investigated 30 mammalian pairs of ligand/receptor for which the first ligand appeared after its receptor in the tree of life, by using common indexes of selection, and proposed different scenarios explaining the earlier appearance of a receptor relative to its ligand. Based on 3D structural studies, our indexes allowed us to classify the evolution of these partners into different scenarios: 1) a scenario where the binding interface of the receptor is already present and under purifying selection before the appearance of the ligand; 2) a scenario where the binding interface seems to have appeared progressively, and 3) a scenario where the binding site seems to have been reshuffled since its appearance. As some scenarios were confirmed by the literature, we concluded that simple indexes can give a good highlight of the evolutive history of two partners that did not appear at the same time. Based on these scenarios, we also hypothesize that the replacement of a ligand by another is a frequent phenomenon during evolution.

## Introduction

The phenotype of organisms results from a set of molecular interactions [1]. For any given phenotype, protein interactions can differ greatly depending on the species. Even between closely related species, many counterparts have differences in affinity and/or number of partners [2]. Even the major vital functions that are homologous between species can be carried out by different protein interactions or mobilized differently depending on the species [3]. For example, the receptor of the natriuretic system is a cyclic GMP receptor in the vertebrate species, whereas it is a cyclic AMP receptor in protostome organisms [4]. Throughout animal evolution, organisms have become more complex [5]. Although certain interactions have been lost, most of them have multiplied, and diversified [6].

The coevolution of interacting proteins and the expansion of the number of partners during evolution is a vast research field [7, 8, 9]. However, understanding how the interaction took place during evolution is a more complex issue. Furthermore, this question can be complicated by the fact that some partner rearrangements can occur during evolution. In two different species, the ortholog receptors can bind different ligands [10, 11, 12]. There are also several cases of orphan receptors, for which it is not known whether the ligand is unknown or does not exist [13, 14, 15]. In the case of nuclear receptors, it was believed that the ancestor of the family was a receptor likely to bind to exogenous inorganic compounds before binding to organic compounds [16], although the possibility of an ancestral endogenous ligand has been demonstrated [17].

Ligands and receptors are part of the molecular pairs that constitute interactive networks. Membrane receptors are the first step to connect the extracellular environment to the cytosol [18]. The ligand and receptor interactions are particularly well documented in human, some of them being involved in human diseases [19, 20, 21]. In a recent study, we demonstrated that most of the genes encoding ligand/receptor partners appeared in the same branches of the animal tree of life [11]. However, that is not the case for all of them. Indeed, in this study, we observed that 210 receptors appeared earlier in phylogeny than their ligands, whereas the opposite case was rarer (116 partners). The present study investigated the scenarios for which ligands appeared after their receptor, not before. In our previous study [22], more than 77 cases in which ligands appeared before their first receptor were described. The cases of ligands appearing first are more difficult to study and access. Part of these ligands appeared very early, as some of them as present in all of the animal tree of life, also in unicellular like S cerevisiae. Moreover, some of them are not proteins encoded by one gene, but the product of synthetic pathways (Dopamine, Serotonin…). Nevertheless, the number of ligands appeared before their first receptor is significantly lower than the reverse phenomenon, indicating that receptors preferentially appeared beforehand.

Understanding how so many receptors were maintained in the phylogeny until the appearance of their ligand is an interesting question, because it would highlight the evolutive strength and dynamism that allow the set up and the evolution of binding partners in the tree of life. Some previous studies have punctually investigated a similar evolutive path of binding partners [23, 24]. However, no study has yet depicted these scenarios on a wider panel of partners.

In the present study, we recovered the partners for which a PDB structure of the ligand/receptor interaction was available in the public databases, and for which the receptors appeared earlier than their ligand(s). We identified 30 pairs, on which we used different indexes to draw the evolutionary and molecular mechanisms that allowed the earlier appearance of receptors. Our approach is a straightforward method to access the global binding pocket set-up scenarios for receptors that appeared before their ligand.

## Methods

### Evolution of the ligand-receptor binding interface

We focussed our study on the human pairs of ligands and receptors, as the available dataset is the most exhaustive. In human, 212 pairs of partners have been identified for which the receptor appeared before the ligand [11]. The differences in the moment of appearance between the ligands and the receptors are highly variable (supp data), as some receptors appeared very early in the animal tree of life, for example first metazoa, and their ligand very later, like in teleost fishes. For each of the 212 pairs of partners, we searched the PDB database [25] for a potential 3D structure of the receptor bound to its ligand. We obtained a set of 30 structures that included the interface between the receptor and its ligand, 22 from humans, 6 orthologous interactions from Mus musculus and 2 orthologous interactions from Rattus norvegicus (supp data).

#### Recovering of the orthologs of the receptors in the animal tree of life

**Fig 1:**
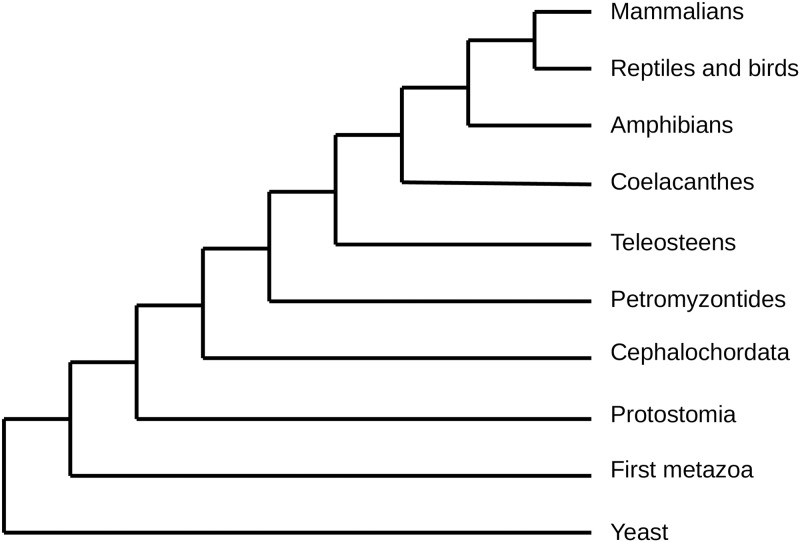
Phylogenetic tree representing the 10 defined branches studied.

For each receptor, the coding DNA sequence was recovered. Its ortholog sequences were searched in all the animal tree of life. The orthologs were detected using the Ensembl phylogenomic trees of life [26]. As previously described [11], ten major taxonomic groups were defined (Fig 1), that correspond to the major branches of divergence in the animal tree of life. These groups will be called the “main branches” in the rest of the text. In each of these groups, when orthologs of the mammalian receptor were present, we recovered the orthologs in a set of species of the branch. For example, if the receptor was present in amniotes, the sequences of its orthologs were recovered in several bird species, snakes, crocodiles, etc, as well as in different mammals.

Given that some taxonomic groups, such as amphibians and chordata, are scarcely represented in Ensembl tree of life, we completed our list of orthologs using the Refseq database [27]. Moreover, a receptor can bind several ligands. As previously described [11], we considered the branch of appearance of the ligands to be the one in which the first ligand appeared.

### Percentage of identity in the binding interface

We hypothesized that the amino acids located at the binding interface of the mammalian receptor sequence might be best conserved in the genomes of the species where the ligand is present, and might be less conserved in the genome of species where the ligand is absent.

For each of the 30 partners, the sequence of mammal receptors and its orthologs were aligned with MUSCLE [28]. In the alignments, the nucleotides corresponding to the amino acids of binding site of the receptor were recovered. For each receptor, the percentage of identity between the mammal site and the binding site of the orthologs was retrieved. Then, we calculated the average rate of similarity of the binding site in the considered branch with the mammal binding pocket. A percentage of 0% means that none of the amino acids composing the human binding pocket is present in the species of the considered branch. A percentage of 100% means that there is no difference between the human binding pocket and the binding site of the ortholog receptors of the species of the considered branch.

These average percentages of identity were compared with the average % ID of the whole sequence, in order to determine whether the binding site was conserved any better than the rest of the sequence.

#### Number of non-synonymous substitutions

We wondered if the binding site of the species of each branch were the same from one to another, independently of their similarity to the mammalian one. A perfect similarity would mean that these amino acids are well fixed in the branch, even if they do not correspond to the mammal binding site, and that the site could have an important function.

In order to do so, we looked at the number of non-synonymous mutations in each branch. The receptors were aligned, and the mutations in the binding sites were studied.

### Evolutionary rates and selection on the binding pocket

We tested the hypothesis that a selective constraint is observed on the binding site in the branches in which the ligand is present, and relaxed in the branches in which the ligand is not present. The indexes of selection and relaxation were tested by site and by branch on the receptors using the package CodeML of the software PAML [29]. For each receptor, the protein sequences of the orthologs were aligned using Pal2Nal software [30]. Then, the binding sites were recovered from the alignments, and aligned specifically. The phylogenetic trees were made using RaxML software [31] using the complete receptors alignments. Positive selection tests were conducted with the CodeML software from PAML [29]. The selection by branch was determined with the model 2 with no clock. A site-based selection was also conducted. To perform the codon-based analysis, we used unrooted trees and selected the option: no clock in the tree. The F3X4 codon matrix was used, and we performed the one ratio-model (model = 0), and NS sites fixed at: one w, nearly Neutral, and positive selection.

Because the phylogenetic distance between species is important (millions of years), we suspected that the results of PAML could be biased. A look at the number of non-synonymous substitutions in the binding pocket of each branch gave us another index, more reliable. The ω ratio is a way of calculating/estimating whether a gene has been subjected or not to positive selection. Thanks to this methodology, we recovered the ω ratio for each site and branches. This index indicates the kind of selection the studied branch or sequence is subjected to. If the ratio is superior to 1, the sequence of branch is subjected to positive selection. A ratio around 1 means a neutral selection. A ratio under 1 means a purifying selection, i.e. the gene is so important for the species that selection avoids any change in the sequence.

All the statistical tests conducted in our study were performed in R, and the simulations and some graphics were performed using python.

## Results and discussion

To gain further insight into this process on the basis of their molecular evolution, we classified the 30 receptor-ligand partners in three scenarios according to the percentage of identity, the ω ratio and the non-synonymous substitution (N). One of the receptor, EPHA3, was not distributed, as its phylogeny failed.

**Fig 2:**
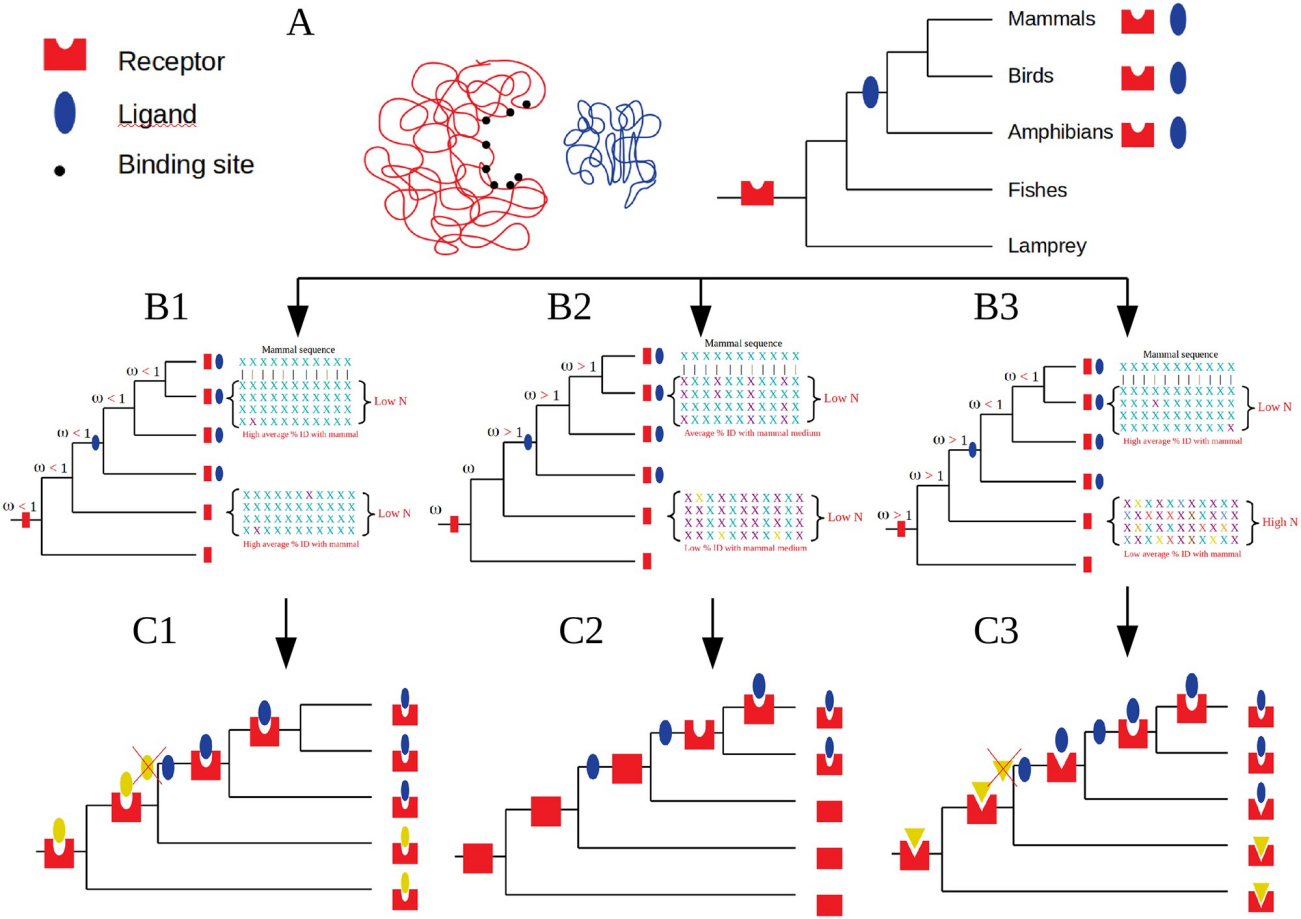
Representation of the three scenarios. A. Receptor binding to its ligand. Left: Schematic representation of the receptor in red with amino-acids of the binding pocket in dark dots, and ligand in blue. As an example, the receptor appeared in vertebrates, and the ligand in tetrapods. B. The N, w, %ID Indexes found in the three scenarios. The X represents the amino acid of the binding pocket. The different colours represent different amino acids in the same position. The N value is the number of non-synonymous substitutions. The alignments represent four sequences of four species of the same branch. In the B1 scenario, almost all the amino-acids of the binding pocket are present in the receptor, even before the appearance of the ligand. In the B2 scenario, there is a low %age ID between the amino-acids of mammal binding pocket with that of species for which the ligand is not yet present, this %age increasing when the ligand is present. In the B3 scenario, there is a low %age ID between the amino-acids of mammal binding pocket with that of species for which the ligand is not then same as the mammal, this %age being very high when the actual ligand “replaced” the first ligand. C. Schematic representation of the 3 different scenarios.

A summary drawing of the three scenarios is shown in Fig 2. The exact values are found in supplemental data.

### Scenario 1

Scenario P1 includes 4 partners (Table 1, Fig 2A, 2B1 and 2C1). For the 4 receptors:

- The ω ratio is always below 1 and very low, in all the branches of the receptor´s tree, independently of the presence of the ligand, being that receptors are always under strong purifying selection.
- The average percentage of identity of the binding pocket to the mammalian receptor´s one, in each branch, is always high (from 100% to a minimum of 40 to 50% in the most divergent branches, Student test, p-values > 0.05, see supp datas).
- The average percentage of identity of the binding pocket to the mammalian receptor´s is higher than the average %ID of the whole sequence, for each branch of the tree (Student test, p-values < 0.05, see supp datas). This can be linked back to the fact that the binding site is always more similar to the mammalian one that the rest of the sequence.
- The number of non-synonymous substitutions in the binding site, among species of the same branches, is always low (inferior to 20), meaning that the amino acids localised in the binding site are conserved within the species of a branch.
- The only amino acids found to be under positive selection are not in the binding pocket.

**Table 1:** Distribution of the receptors in their 3 scenarios.

Scenario 1	Scenario 2	Scenario 3
ADIPOR1	AXL	CCR5
ADIPOR2	CSF2RB	CXCR4
AGTR1	IGF2R	EPHA5
TNFRSF21	IL1R1	EPHA7
	ITGA4	FSHR
	NTSR1	GCGR
		GFRA1
		GLP1R
		HCRTR1
		HCRTR2
		IFNAR2
		KIT
		LPHN3
		NPR1
		OPRL1
		OPRM1
		RXFP2
		SORT1
		TSHR

Taken together, these indexes support the assumption that the binding site was already present and functional since the birth of the gene encoding the receptor. As the mammalian ligand appeared later in evolution, we could draw up a scenario in which an ancestral ligand, phylogenetically unrelated to the human one, but very similar to it in its binding properties, was replaced in some branches, including the mammals [32]. Such scenarios have already been described [33, 34]. Indeed, thanks to these examples, we understand that for these partners, the receptor in fact did not appeare before its first ligand. Rather, the receptor probably appeared concurrently to its first ligand, and during evolution, there was a convergence of ligand-receptor due to the recruitment of a novel ligand. In this scenario, we found the human ADIPOR1 receptor, which binds adiponectin. The literature confirms this scenario. Its yeast, its ortholog PAQR3, was considered an orphan receptor. However, it has been shown experimentally to be able to bind human adiponectin in vitro, using the same binding pocket as its human ortholog [35]. A PAQR3 ligand is still unknown in yeast, but the presence of an adiponectin-like peptide was demonstrated in insects [36]. However, this insect ligand is phylogenetically unrelated to the mammalian one. A similar scenario was demonstrated by Bridgham et al, [37], who showed that the aldosterone receptor first acquired the ability to bind aldosterone 50 Mya before aldosterone even appeared, at a time when an initial ligand structurally similar, but distinct from aldosterone, existed.

The evolutionary scenario of recruitment of novel ligand can be due to the evolutive convergence of independent ligands [37]. This was documented for several molecules such as defensins [38], serine and cysteine [39].

### Scenario 2

Scenario 2 includes 6 partners (Table 1, Fig 2A, 2B2 and 2C2). For the 6 receptors:

- The ω ratio is seen to be under positive selection (superior to 1) in at least one of the branches in which the ligand is already present, or in most of them. Even in the presence of the ligand, the binding site was submitted to selective pressure.
- The average percentage of identity of the binding pocket to the mammalian receptor is very low in the branches in which the ligand is not present (between 0 to 30%). In the branches in which the ligand is present, this percentage increases and is high in the mammal branch (Student test, p-values < 0.05, see supp datas).
- The average percentage of identity of the binding pocket to the mammalian receptor´sis never higher that the average %ID of the whole sequence, for each branch of the tree. The binding site is never more similar to the mammal one than the rest of the sequence (Student test, p-values > 0.05, see supp datas).
- The number of non-synonymous substitutions in the binding site, between species of the same branches, is low (inferior to 20), meaning that the binding site is conserved between the species of a given branch. However, the previous index indicates that these conserved amino acids are not the same as the mammal ones in the branches without the ligand. Moreover, the number of non-synonymous substitutions in the branches without any ligand is not different than the number in the branches in which the ligand is present. One exception for ITGA4.
- The amino acids undergoing positive selection (in IL1R1 and NTSR1) are not involved in ligand binding.

In this scenario, the binding site is not present in the species in which the ligand is not. We could thus suspect that the ancestral receptor did not possess that binding site. Moreover, the fact that some branches in which the ligand is present have a binding pocket under positive selection could mean that the appearance of the ligand exerts a positive pressure on fixation of a binding pocket. These elements seem to indicate that the binding site was not present at the time of the appearance of the ligand in the common ancestor.

In keeping with this scenario, some cases of ligand binding acquisition have already been documented. For example, the FXR receptors (farnesoid X receptor) are nuclear receptors which bind bile salts in mammals. The ability of FXR to bind such ligands is not present in shark, suggesting that, unless it has been lost in sharks, it appeared during vertebrate evolution by molecular exploitation [40]. It was also demonstrated that a GBR2 (metabotropicy-aminobutyric acid b receptor) has a function which does not involve any receptor activity [41]. Such examples suggest that some molecules anchored in the membrane could have a primary function before acquiring the ability to bind a ligand and becoming membrane receptors.

Interestingly, we find the IGF2R receptor (Insulin-like growth factor 2 receptor) in this category, for which none of the amino acids present in the human binding pocket are present in the non-mammalian species. The ω ratios were particularly interesting. We observed a ω ratio superior to 1 in the branches of protostomians and chordates. Then, in teleost, the ω ratio become inferior to 1. But in mammals, the ω ratio increases again and is higher than 1. In humans, IGF2R enables intracellular traffic of lysosomal enzymes, and internalizes IGF2 for degradation [42]. In agreement with our results, although the sequence of IGF2 is well-conserved in vertebrates, it has been demonstrated that the ability for IGF2R to bind IGF2 was acquired only in mammals after the divergence with the monotremes [43], due to the evolution of the binding pocket in this group [44].

### Scenario 3

Scenario P3 includes 19 partners (Table 1, Fig 2A, 2B3 and 2C3). For the 19 receptors:

- The ω ratio is under strong purifying selection in the branches in which the ligand is present. However, in the branches in which the ligand just appeared, the ω ratio is superior to 1. That means that positive selection, and so redesigning of the binding pocket is observed for the period corresponding to the appearance of the ligand. For most of them, the ω ratio is also higher than 1 in the branches in which the ligand has not yet appeared.
- The average percentage of identity of the binding pocket to the mammalian receptor´s is very low in the branches in which the ligand is not present (between 0 to 30%). This percentage increases starting from the first branch in which the ligand is present and becomes very high as it reaches the mammalian branch (Student test, p-values < 0.05, see supp datas).
- The average percentage of identity of the binding pocket to the mammalian receptor´s is not different, or even lower, than the average %ID of the whole sequence in the branches in which the ligand is not present (Student test, p-values > 0.05, see supp datas). On the contrary, this percentage becomes equal to or higher than the rest of the sequence in the branches in which the ligand is present (Student test, see supp datas).
- The number of non-synonymous substitutions in the binding site, between species of the same branches, is always significantly higher in the branches in which the ligand is not present than in the branches in which the ligand is present.
- The amino acids under positive selection are not involved in ligand binding.

This scenario is quite similar to the previous one, because all the indexes suggest that the binding site is not present in the branches in which the ligand is absent. However, the fact that the binding site is often under positive selection in the branches that do not possess the ligand, and that this positive selection can also be observed at the appearance of the ligand, suggests that the binding site underwent specific reshuffling during evolution. We suggest that the ancestral receptor could already have had a binding site in that position, but completely different from the mammalian one. The ancestral pocket would have bound to other different ligands. Then, throughout evolution, the receptors specialised in the binding of newly appeared ligands.

In this scenario, the replacement of the ligand would be due to a conformational change of the receptor binding pocket. In confirmation to that hypothesis, in our database, we found the receptor FSHR (Follicle Stimulating Hormone Receptor). This receptor appeared in Eumetazoa. The ligand FSH appeared later in vertebrates. The FSH receptor (FSHR) ortholog in insects binds a ligand named FSH-like with a similar signalling pathway [23, 24]. Furthermore, the FSH-like molecules in these invertebrates are not phylogenetically related to the vertebrate FSH [45]. This means that the ancestor of FSHR and the ancestor of FSH appeared respectively in the metazoa and protostomes, but that they were able to bind to each other only in vertebrates. Because the FSHR binds FSH-like molecules in invertebrates and FSH in vertebrates, both ligands being different and not phylogenetically related, this suggests an important change in the binding pocket of the initial FSHR receptor during evolution.

**Fig 3:**
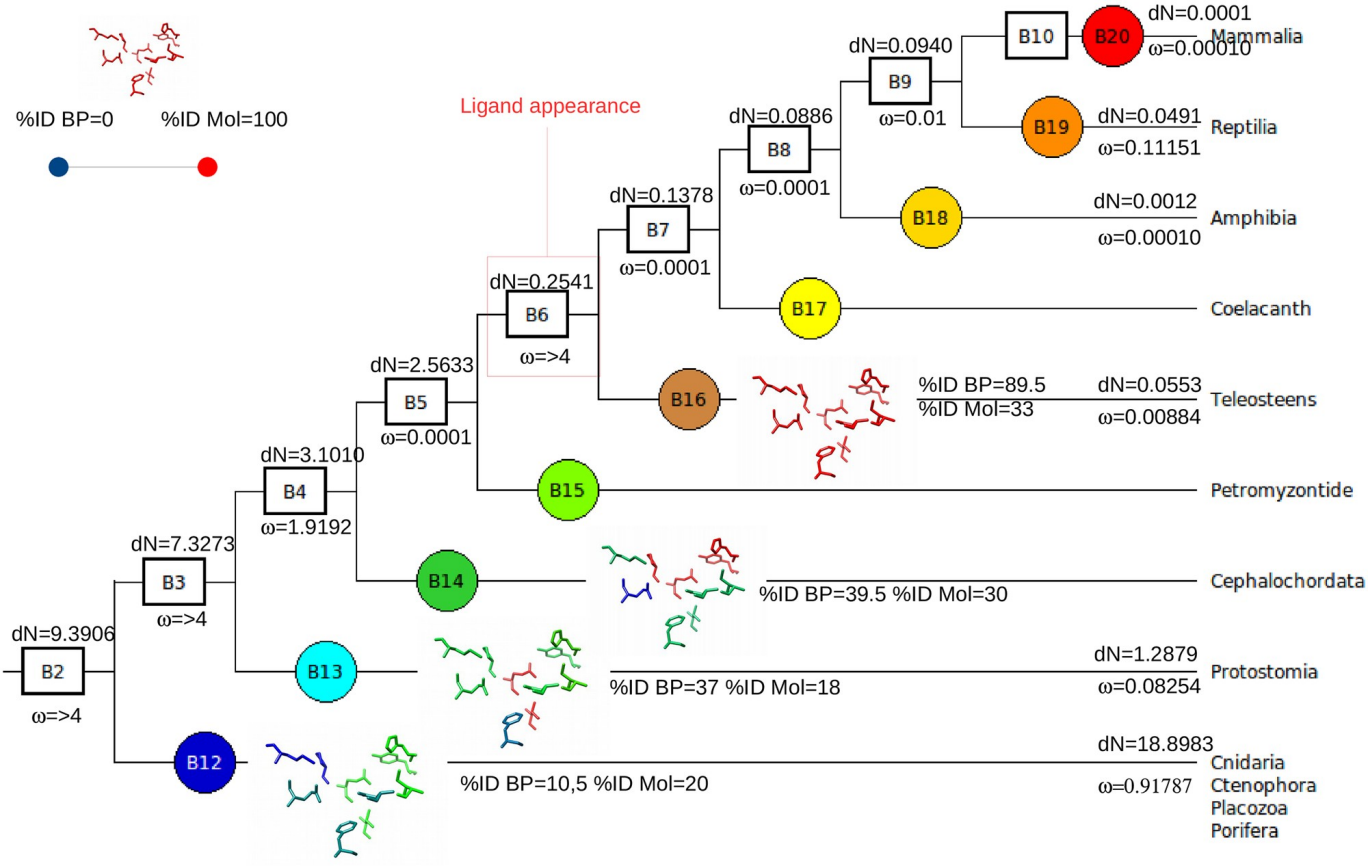
Example of a receptor of the scenario 3, HCTR1. In such an example, are represented all of the indexes obatined for HCTR1, in its phylogenetic context. The cladogram represents the branches in which the receptor was found. B2 to B10 represent the phyla to the Families in which the receptor is present: B2. Metazoans, B3. Bilaterians, B4. Deuterostomians, B5. Vertebrates, B6. Teleosts, B7. Sarcopterygians, B8. Tetrapods, B9. Amniotes to B10. Mammals. B11 to B20 represent the internal branches. The gene encoding HCTR1 appeared in the first metazoan. The % ID BP corresponds to percentage of identity of the binding pocket of the species of the internal branch compared to the mammal binding site alignment; The % ID Mol is the percentage of identity of the molecule of the species of the internal branch compared to the mammal sequence in the alignment. The calculated ω ratio corresponds to the ω ratio of the binding pocket only, calculated by branch. The dN is the number of non synonymous substitutions divided by the number of non synonymous sites for the species of the internal branch. The conserved binding sites were recovered from the 3D structures of the receptors. The amino acids were colored according to their %ID with the mammal coresponding one. The color chart, from blue, to green, orange and red corresponds to a progressive increase of the % ID of the binding pocket with that of the mammal, until reaching values close to 100, here when the ligand appears (branch 16 teleosts).

In this scenario, we could also hypothesize that the receptor, during evolution, adapted to different ligands. In this Scenario, we found the HCRTR1 (hypocretin receptor). According to the literature, the ancestor of the gene encoding HCRTR1 appeared in the first metazoa. Despite the appearance of its first ligand in vertebrates, we observe that the percentage of identity of the binding interface is already high in chordates. Moreover, the number of non-synonymous substitutions inside the branches and the ω decrease in the branches before the appearance of the ligand. Indeed, there should exist a pressure for the conservation of these residues in the binding pocket once they have appeared. Such a pressure could be due to the presence of another ligand before the branch of appearance of the human ligand (here, the vertebrates). In line with this expectation, Yun et al, 2015, found that the HCRTR1 ortholog in *Ciona intestinalis* has a functional ligand different than the mammal one. Why the ligand was replaced remains to be clarified. The indexes of HCRTR1 are shown in Fig 3.

## Conclusions

By investigating 30 cases with known PDB structures, we show that several scenarios can be proposed. In most of the scenarios presented above, we suggest that the ancestral receptor was able to bind an ancestral ligand, and that the origin of the ligands can differ among the branches. Therefore, as stated by Bridgham [37] and Kachroo [46], we concur that in the case of receptors that appeared before their ligand, the coevolution between an ancestral ligand and the receptor is a driving force that operates during the evolution of binding partners. We distinguished two different scenarios for receptors that appeared before their ligand: either the second ligand replaced the first by adopting a similar binding structure, or a change in the binding pocket of the receptor gave rise to a change of ligand partner. Interestingly, the scenario 2 also suggest that some receptors could have appeared without binding ability and have acquired it during evolution. A hypothesis for the receptors that do not have any amino acids in common with the mammalian binding pocket is that the common ancestor was an orphan receptor. Understanding whether an orphan receptor is the ancestral form of the nuclear receptors is still currently a matter for debate [13]. However, the nuclear orphan receptors have crucial functions [14], and can act without specific ligands [47]. We could then suppose that some of the current receptors that work with a ligand acquired an "official" binding partner during evolution.

In this study, we only consider the ligand and its receptor as a simple entity in which the presence of both partners allows a biological process, that is the transmission of an intracellular signal in the cell. However, ligands and receptors are in fact part of a somewhat more complex interactive network, with much more components that only two partners. In our study, indexes tend to show that the mammalian receptors for which the current ligand appeared later during evolution had in fact an ancestral ligand with which it interacted. However, in future studies, emphasis should be put on studying the other potential binding sites of the receptors that could be activated by allosteric ligands. In fact, molecules other than the endogenous ligands, that would bind to other binding site(s), can activate the dimerization of the receptor and act on the intracellular signaling [48]. Cases of receptors activated by other receptors can also be observed (ex GFRA1 activating RET receptor).

## Supporting information

S1 Data (ODS)

S2 Data (ODS)

S3 Data (ODS)

S4 Data (ODS)

S5 Data (ODS)

S6 Data (ODS)
